# Candidate pigmentation genes related to feather color variation in an indigenous chicken breed revealed by whole genome data

**DOI:** 10.3389/fgene.2022.985228

**Published:** 2022-11-21

**Authors:** Huie Wang, Junhui Wen, Haiying Li, Tao Zhu, Xiurong Zhao, Jinxin Zhang, Xinye Zhang, Chi Tang, Lujiang Qu, M. Gemingguli

**Affiliations:** ^1^ Xinjiang Production and Construction Corps, Key Laboratory of Protection and Utilization of Biological Resources in Tarim Basin, Tarim University, Alar, China; ^2^ College of Life Science and Technology, College of Animal Science and Technology, Tarim University, Alar, China; ^3^ National Engineering Laboratory for Animal Breeding, Department of Animal Genetics and Breeding, College of Animal Science and Technology, China Agricultural University, Beijing, China; ^4^ College of Animal Science, Xinjiang Agricultural University, Urumchi, China

**Keywords:** plumage colors, genome, chicken, pigmentation genes, genetic marker

## Abstract

Chicken plumage color is an inheritable phenotype that was naturally and artificially selected for during domestication. The Baicheng You chicken is an indigenous Chinese chicken breed presenting three main feather colors, lavender, black, and yellow plumages. To explore the genetic mechanisms underlying the pigmentation in Baicheng You chickens, we re-sequenced the whole genome of Baicheng You chicken with the three plumage colors. By analyzing the divergent regions of the genome among the chickens with different feather colors, we identified some candidate genomic regions associated with the feather colors in Baicheng You chickens. We found that *EGR1*, *MLPH*, *RAB17*, *SOX5*, and *GRM5* genes were the potential genes for black, lavender, and yellow feathers. *MLPH*, *GRM5*, and *SOX5* genes have been found to be related to plumage colors in birds. Our results showed that *EGR1* is a most plausible candidate gene for black plumage, *RAB17*, *MLPH*, and *SOX5* for lavender plumage, and *GRM5* for yellow plumage in Baicheng You chicken.

## Introduction

Chicken plumage color is driven by natural, sexual, and artificial selection (Roulin and Ducrest, 2013). The diversity of the plumage colors is regulated by multiple genes, and a number of genetic mutations have been identified for certain feather color traits (Cieslak et al., 2011). Multiple genes have been found to be associated with animal pigmentation (http://www.ifpcs.org/colorgenes/) (Baxter et al., 2019). Given the complexity of pigmentation traits, the genetic foundation of some of them still remains unraveled.

Feather colors in birds are mainly determined by the distribution and quantity of eumelanin and phaeomelanin and the size, number, and type of compartments containing melanin in keratinocytes. Two types of melanin are produced by neural crest-derived melanocytes (Moreiras et al., 2021). The distribution of melanosomes within melanocytes is the result of competition between microtubules and actin-dependent transport (Gross et al., 2002). Melanosomes must be transported from the perinuclear region to melanocyte dendrites and then to keratinocytes (Alzahofi et al., 2020; Jiang et al., 2020). Some studies have shown that melanin-based color phenotypes are associated with mutations in melanin-producing genes. For example, melanocortin 1-receptor (*MC1R*), a seven-transmembrane helix-bearing G protein-coupled receptor on melanocytes, plays a crucial role in determining the melanin type and underlies the pigmentation traits in a wide range of animals. In chickens (Kerje et al., 2003; Takeuchi et al., 1996), Japanese quail (Nadeau et al., 2006), pigs (Fang et al., 2014), and horse (Ludwig et al., 2009), the extended black (E) locus controls coat color and pattern, and the molecular basis of its actions is rooted in the MC1R gene (Schiöth et al., 2003).

The Baicheng You chicken is an indigenous Chinese breed, and its main feather color types are black, yellow, and lavender. To identify the genetic foundation of the plumage colors, we employed a genome-wide selective sweep (Pavlidis et al., 2013) analysis by comparing chicken genomes with respect to the three colors to identify potential regions that are differentially selected for feather color (Chen et al., 2010; Pavlidis et al., 2013). Our results identified candidate genes related to the three types of feather colors in Baicheng You chicken.

## Materials and methods

### Chicken sampling

**FIGURE 1:**
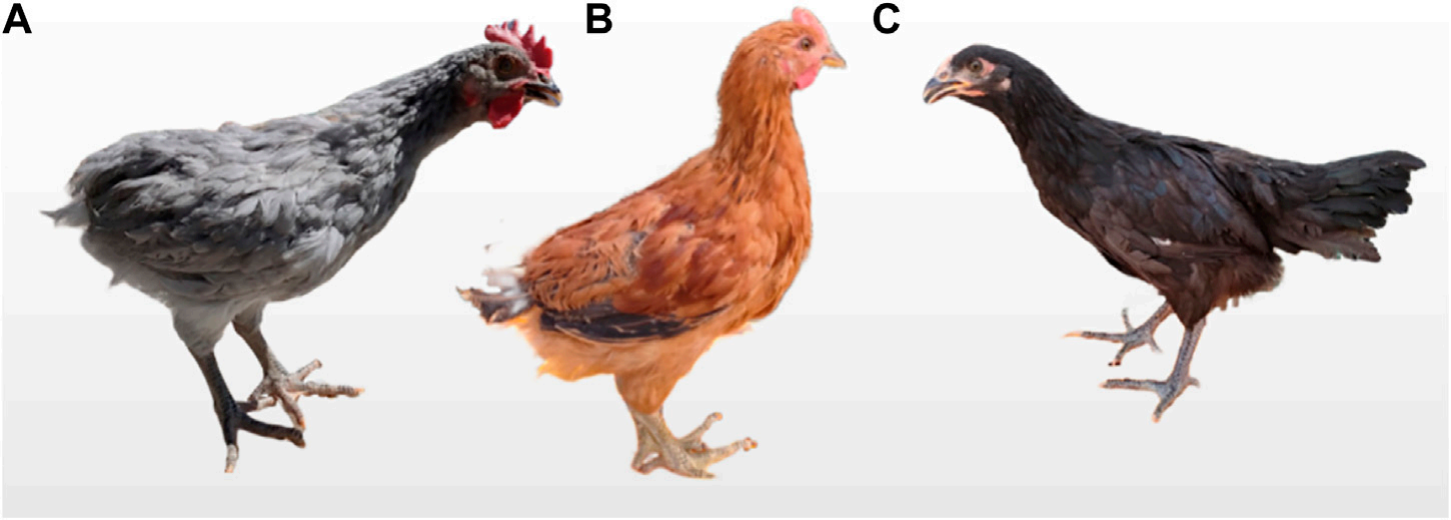
Baicheng You chicken with black **(C)**, yellow **(B)**, and lavender **(A)** feather colors.

We used a local Chinese Baicheng You chicken population to screen candidate genes for feather colors. First, 30 adult chickens were selected from differentiated sub-populations selected for the three target feather colors (Figure 1), lavender, black, and yellow, with 10 samples for each sub-population (ADMIXTURE and PCA; Supplementary Figure S1). Blood samples were obtained from the brachial veins by standard venipuncture and were placed into centrifuge tubes containing an anticoagulating agent.

### Genomic resequencing and variant calling

Genomic DNA was extracted from the 30 chickens using the standard phenol/chloroform method, and random interruptions were made using a Covaris ultrasonic crusher. We performed 150-bp paired-end re-sequencing using the Illumina HiSeq 2500/NovaSeq 6000 system according to the manufacturer’s protocols. We used fastp (v0.20.0) software to filter residual primers, adapters trimming, and lower-quality reads, and used the -q 20 -u 30 parameters (from the Fastp manual, q, --qualified_quality_phred indicate the quality value that a base is qualified, thus default 15 means phred quality ≥ Q15 is qualified) for the quality control of the 150-bp paired-end raw reads (Chen et al., 2018). If the quality value of the per-base or per-read is less than 20, this is considered to indicate low quality. If the percentage of low-quality bases is 30%, the reads with too many N bases are excluded. If the content of N bases is greater than n, the read/pair will be discarded, and the default value is 5, such as when one has a high quality score (>Q30) and the other has a low quality score (<Q15). To reduce false corrections, fastp only performs a correction if the total mismatch is below a given threshold T (T ¼ 5) (Chen et al., 2018). The clean reads were mapped to the chicken genome (https://www.ncbi.nlm.nih.gov/, assembly: GCA_016700215.2) using BWA-MEM (v0.7.17) with default parameters, except for -t 4 -R (-t is thread, -R is set the reads header, and it is split with \t) option (Li et al., 2008). We sorted the alignment bam files using the Samtools (v-1.11) software package, and duplicate reads were removed using the picard (http://broadinstitute.github.io/picard/) tools MarkDuplicates. Genome Analysis Toolkit (GATK,v-4.2) (https://gatk.broad institute.org/) was then used for downstream processing and variant calling. Using the HaplotypeCaller algorithm of the GATK pipeline in the genomic Variant Call Format (gVCF), we obtained the genotype likelihoods at each site in the reference genome for each individual. We called the SNPs and indels using the HaplotypeCaller algorithm. Individual gVCF files were then merged into a multi-individual VCF using the GenotypeGVCFs tool of the GATK pipeline. It is imperative to filter raw SNP candidates in the genotyping workflow because it allows the shrinking of false-positive calls due to biases in the sequencing data (Jiang et al., 2021; Xiangtao Liu et al., 2013). We called SNPs and indels using the SelectVariants algorithm. Next, these variants were used as input for hard filtering in the GATK pipeline based on six statistics to identify SNPs or indels: QUAL < 30.0; QualByDepth (QD) < 2.0; FisherStrand (FS) > 60.0; RMS MappingQuality (MQ) < 40.0; MappingQualityRankSunTest (MQRankSum) < -12.5; and ReadPosRankSumTest (ReadPosRankSum) < -8.0 (Li et al., 2008). ClusterWindowSize and clusterSize were set to 10 and 3, respectively. Generally, consecutive SNPs tend to provide a high proportion of false-positive results in terms of selection signatures associated with plumage color (Margot E. Bowen et al., 2011).

### Selection signature analysis for plumage colors

The cross-population composite likelihood ratio (XP-CLR) method is mainly based on the gene Site Frequency Spectrum (SFS) principle, which is based on the difference in multilocus allele frequency between two populations and is used in selection signal detection (Maria Ines Fariello 2013; Chen et al. 2010). These methods have been widely used to discover the loci and genes related to difference in traits, such as hair length in Tianzhu white yaks (Bao et al., 2022), body size in dogs (Lyu et al., 2021) and ponies (Asadollahpour et al., 2020), and meat quality in Angus cattle (Taye et al., 2018). We used XP-CLR (v1.1.2) (Chen et al., 2010) statistical methods to detect genetic differentiation in Baicheng You chicken plumage colors (Pavlidis et al., 2013). The values of XP-CLR were calculated using the python script XPCLR, downloaded from Github (https://github.com/hardingnj/xpclr). The corresponding parameters were set as follows: maximum SNPs = 600, ld values = 0.95, window size = 40,000 (Lee et al., 2018). The regions with XP-CLR values in the top 1% were considered candidate regions, and genes with overlapped candidate sweeps were considered candidate genes. Six feather color models were used to identify candidate direct selection regions: 1) black *vs.* lavender; 2) black *vs.* yellow; 3) yellow *vs*. lavender; 4) black *vs*. yellow + lavender; 5) lavender *vs.* black + yellow; and 6) yellow *vs*. black + lavender. In addition, the genes were annotated using the chicken genome (https://www.ncbi.nlm.nih.gov/, assembly: GCA_016700215.2) and NCBI databases. Variant effect predictor (VEP) was used to annotate gene variants according to their functional categorization, including the following categories: up- and downstream gene, frameshift, 3- and 5-prime UTR, synonymous, missense, start lost, intronic, and splice region. We used the R qqman package to create a Manhattan plot of XP-CLR for the association analysis.

## Results

### Sequencing and variations

After quality control, a total of ∼8.9 million high-quality paired-end reads were obtained from the 30 chickens (267.73 Gb clean base). The average sequencing depth per individual was 8.11×, and the average genome coverage was 98.62%.

A total of 18.37 million SNPs and 2.61 million indels were retained for the association analysis. In addition, where more than 3 SNPs were clustered within a 10-bp window, they were all considered false positives and deleted. In the subsequent analyses, we used only biallelic SNPs on the chromosomes.

### Selection signature analysis for plumage colors

**FIGURE 2:**
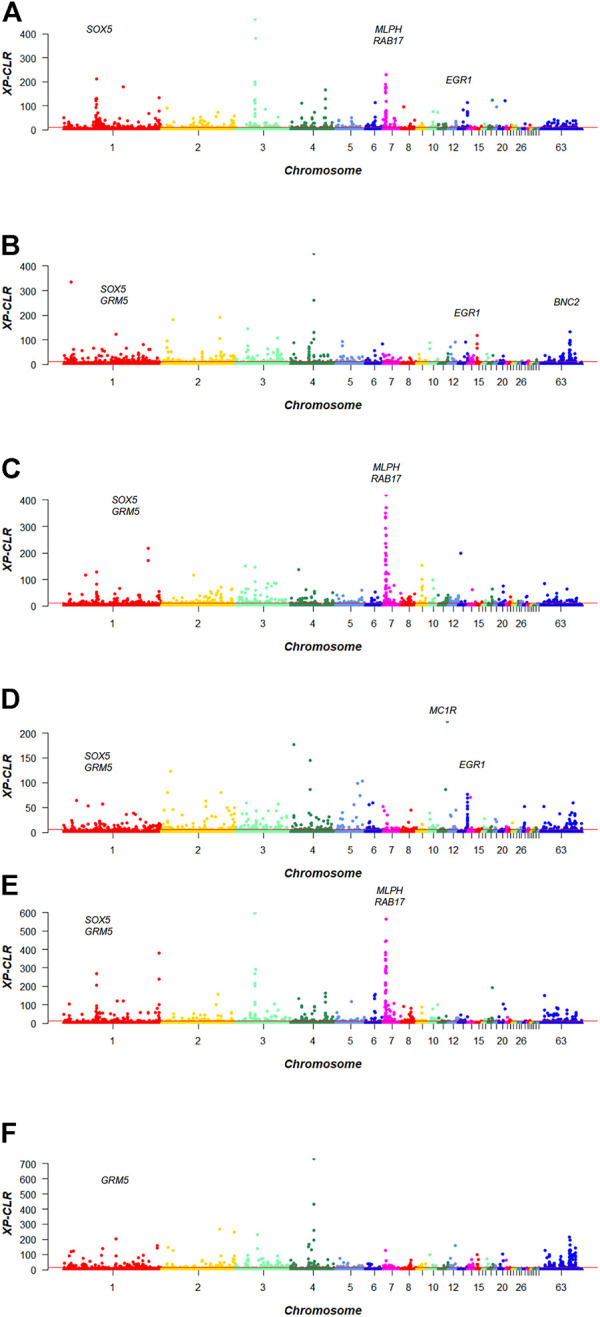
XP-CLR Mahattan plot of plumage colors in Baicheng You chicken. **(A)** Black *vs*. lavender; **(B)** black *vs.* yellow; **(C)** lavender *vs.* yellow; **(D)** black *vs.* lavender + yellow; **(E)** lavender *vs*. black + yellow; **(F)** yellow *vs.* black + lavender.

The candidate genes for the three kinds of feather colors were identified using XP-CLR statistical methods in Baicheng You chicken. The total numbers of genes with positive selection signatures detected in the six groups were as follows: 934 genes (black *vs*. lavender), 3620 genes (black *vs*. yellow), 989 genes (yellow *vs*. lavender), 685 genes (black *vs*. yellow + lavender), 836 genes (lavender *vs*. black + yellow), and 820 genes (yellow *vs*. black + lavender), including cell growth, differentiation, migration, and immune regulation (Supplementary Table S2–S7). XP-CLR scores are presented in Figure 2.

First, to obtain the genes associated with black plumage, the scanning regions of top 1% of black *vs*. lavender, black *vs.* yellow, and black *vs*. yellow + lavender models were extracted, respectively (Figures 2A,B,D), and the intersection of the three sets of data was taken. We obtained the overlap windows at chromosome 13: 18940001–18980000 bp, which overlapped with *EGR1* gene.

Second, we extracted top 1% of sweep regions of black *vs.* lavender, lavender *vs.* yellow, and lavender *vs.* black + yellow models, respectively (Figures 2A,C,E), and the same method was used to obtain the intersection of the three groups of data. One window was found at chromosome 7: 4800001–4840000 bp, harboring *RAB17*, and we also identified a window at chromosome 7: 4820001–4860000 bp, containing *MLPH*. Another window was located at chromosome 1: 65940001–65980000 bp, which overlapped with the *SOX5* gene. We thus obtained *MLPH*, *RAB17*, and *SOX5* as genes that could be associated with lavender plumage color.

We detected the top 1% of sweep regions of black *vs*. yellow, lavender *vs*. yellow, and yellow *vs*. black + lavender models, respectively (Figures 2B,C,F), and again took the intersection of three sets of data. The overlap window at chromosome 1: 18240001–18280000 bp, containing the *GRM5* gene.

The lavender phenotype has been found to be related to *MLPH* and *Rab17* (Roulin and Ducres, 2013). *SOX5*, as a transcription factor, indirectly regulates the synthesis and transport of melanocytes (Min et al., 2022; Stolt et al., 2008; Wang et al., 2016). *EGR1* is expressed in mouse hair follicles (Mcmahon et al., 1990). *GRM5* is a candidate gene in the Polverara chicken (black *vs*. white) (Mastrangelo et al., 2020). Thus, the results showed that *MLPH*, *RAB17*, *SOX5*, *EGR1*, and *GRM5* genes were associated with lavender, black, and yellow plumage colors in Baicheng You chicken.

### Variations in candidate genes

A total of 258 SNPs and 28 indels were identified in *MLPH* (Supplementary Table S8). In the coding regions of the *MLPH* gene, we detected eight synonymous, two non-synonymous nucleotide substitutions, and one deletion. Interestingly, we found an A99G substitution in exon 1 and one deletion (AG861A) in exon 6 that were present in lavender feather chickens.

*RAB17* possessed a total 179 SNPs and 6 indels (Supplementary Table S9). Five synonymous and four non-synonymous nucleotide substitutions were detected in the coding region of *RAB17* gene, and G514A (Val172Ile) substitution in exon 5 appeared in lavender plumage color (Table 1).Three synonymous and two non-synonymous nucleotide substitutions were detected in exon 2 of the *EGR1* gene (Supplementary Table S10).

**TABLE 1:** Candidate genes based on the SNPs and amino acid substitutions in chicken with black/lavender/yellow plumage .

Feather	Candidate gene	Location	Exon/intron	CDS_position	Protein_position	Existing_variation
Lavender	MLPH	chrom 7: 4833504	Exon 1	A99G	Leu33	—
	—	chrom 7: 4826596	Exon 6	AG861A	Tyr287X	—
	RAB17	chrom 7: 4811426	Exon 5	G514A	Val172Ile	rs312298254
	SOX5	chrom 1: 65940001–65980000	—	—	—	—
Black	EGR1	chrom 13: 18951764	Exon 2	T1297C	Ser433Pro	rs317658682
Yellow	GRM5	chrom 1: 189315001–189325000 bp	Intron	—	—	—

The remaining two plumage color loci at chromosome 1 exhibited the strongest association signals within the intronic regions of *GRM5* and *SOX5*, and they are obvious candidates for controlling normal variation in chicken plumage colors (Supplementary Tables S11, S12). However, *GRM5* lies 33.82 kb upstream of *TYR*, which is also involved in pigmentation, which could affect pigmentation by regulating the expression of *TYR* (Sandra Beleza et al., 2013; Smyth et al., 2006). Therefore, we hypothesized that the non-coding DNA variation of *GRM5* and *SOX5* might affect pigmentation by regulating the expression of neighboring genes or influence itself expression by regulating the enhancer activity of the genes themselves. Candidate genes based on the SNPs and amino acid substitutions in black, lavender, and yellow feathers in Baicheng You chicken are summarized in Table 1.

## Discussion

### Lavender plumage

In our study of the three feather colors of Baicheng You chicken, we focused specifically on the loci that may play a more important role in the evolution of feather color through natural, sexual, and artificial selection. Here, we used comparative genome analysis between black, yellow, and lavender sub-populations to identify the genetic basis underlying the variation in feather color among Baicheng You chicken. In this study, several previously reported genes were found to be involved in lavender feather-related traits, such as MLPH, RAB17, and SOX5. In previously published articles, non-synonymous mutation (c. 103 C>T) (Vaez et al., 2008) and (c. 1909 A>G) (Xu et al., 2016) of *MLPH* resulted in the development of the lavender or light-gray plumage color phenotype in chicken. In this study, we did not find SNP (c.1909 A>G), but a frameshift deletion (c. 861 indel G) was located at exon 6 of *MLPH*, which leads to a completely different protein. Vaez *et al.* found that the absence of exon 6 in *MLPH* gene transcript in lavender phenotype chickens (Vaez et al., 2008), and frameshift deletion in exon 5 of *MLPH* gene has similarly been reported in rabbit (*Oryctolagus cuniculus*) breeds (Fontanesi et al., 2014). Deletions have also been found in leaden mice and cat (Ishida et al., 2006). The variations in *MLPH* gene could cause transport defect melanosomes in melanocytes, resulting in the dilution of coat or plumage colors in cats (Ishida et al., 2006), dogs (Bauer et al., 2018; Drögemüller et al., 2007; Van Buren et al., 2020), cattle (Li et al., 2016), sheep (Posbergh et al., 2020), rabbits (Demars et al., 2018; Jia et al., 2021), and minks (Cirera et al., 2013), as well as Griscelli syndrome type-3 in humans (Al-Mousa et al., 2016; Çağdaş et al., 2012; Gironi et al., 2019; Jo et al., 2020; Ménasché et al., 2005). McMurtrie *et al.* reported that *RAB17* is an attractive candidate for leaden mice, though failed to identify any *RAB17*-coding region mutations, because it was expressed in polarized epithelial cells (E. B. McMurtrie et al., 1997). A non-synonymous substitution (G514A) in exon 5 of *RAB17* gene by whole genome re-sequencing was detected, resulting in change in the amino acid Val172Ile in this study.

The transcription factor *SOX5* has been detected in the early migrating neural crest of chicken (Perez-Alcala et al., 2004). There are two explanations for the regulation of transcription factor *SOX5* on melanocytes. A 7.6-kb non-coding deletion near *SOX10* was found to be associated with pale yellow feathers (Zhu et al., 2022), and *SOX5* played a role in melanocyte lineage by regulating the activity of transcription factor *SOX10* (Min et al., 2022). The other was up-regulation of the expression of *TYR* through *MITF*, thereby reducing the synthesis of melanin (Wang et al., 2016).

### Black plumage

In chicken, *MC1R* is a single exon gene without intron on chromosome 11, and it is associated with the traditional feather color Extension locus (E). Although *MC1R* is among the genes that have been extensively studied for their association with feather color, we did not identify it except for in the black *vs*. yellow + lavender model. We had the major gene *EGR*1 of black plumage by XP-CLR algorithm, and we hypothesized that the variation loci T1297C (Ser433Phr) in *EGR1* could be associated with the synthesis of eumelanin. *EGR1*, a member of the immediate early family, is an important nuclear transcription factor (Gómez-Martín et al., 2010). *EGR1* mRNA was highly expressed in hair follicles in the process of mouse embryogenesis (Mcmahon et al., 1990). *EGR1* could also up-regulate the expression of *TYR* by binding *MC1R*, leading to the synthesis of large amounts of eumelanin.

### Yellow plumage

In this study, *GRM5* was associated with yellow plumage color. Our results coincided with those of Mastrangelo et al. (2020). In recent years, studies of human skin and eye color have found that the skin color loci at 11q14.3 is strongly correlated with the intronic regions of *GRM5*, and non-synonymous mutation in *TYR* (S192Y) is in linkage disequilibrium with the most significant SNPs in *GRM5* (Sandra Beleza et al., 2013). Kaustubh Adhikari *et al.* have also demonstrated the presence of multiple independent signals of association in *GRM5/TYR* (Adhikari et al., 2019; Miao et al., 2017). *GRM5* may affect pigmentation by regulating the expression of *TYR*.

## Conclusion

In our study, we showed that *MLPH*, *RAB17*, *SOX5*, *EGR1*, and *GRM5* were candidate pigmentation genes in Baicheng You chicken breed, using whole genome data. Notably, mutations of *MLPH* (AG861A), *RAB17* (G583A), and *SOX5* have been found to be related to lavender plumage. *EGR1* (T1297C) was a most plausible candidate gene for black plumage, and *GRM5* for yellow plumage in Baicheng You chickens. Overall, the candidate genes identified herein could help elucidate the structure and composition of the genome underlying plumage colors and provide novel insights into the regulation mechanisms of feather color development in Baicheng You chicken, as well as other chicken breeds.

## Data Availability

The datasets presented in this study can be found in online repositories. The names of the repository/repositories and accession number(s) can be found in the article/Supplementary Material.
